# Improving the Surface Color and Delaying Softening of Peach by Minimizing the Harmful Effects of Ethylene in the Package

**DOI:** 10.3390/foods14142472

**Published:** 2025-07-15

**Authors:** Hongsheng Zhou, Siyu Ma, Jing Zhao, Ying Gao, Wen Huang, Yingtong Zhang, Jun Ling, Qian Zhou, Pengxia Li

**Affiliations:** 1 Jiangsu Academy of Agricultural Sciences, Nanjing 210014, China; zhouhongsheng1234@163.com (H.Z.); 20180002@jaas.ac.cn (Y.Z.); robinlingjun@126.com (J.L.); 2 Department of Food Science, Shenyang Agricultural University, Shenyang 110866, China; 13842063029@163.com (J.Z.); 15148272494@163.com (Y.G.); 3 School of Food and Biological Engineering, Jiangsu University, Zhenjiang 212013, China; msy1126@yeah.net; 4 Nanjing Institute of Vegetable Science, Nanjing 210042, China; xiaohuangwen588@163.com

**Keywords:** peach, modified atmosphere packaging, ethylene absorbent, firmness, anthocyanin

## Abstract

Peach is a typical ethylene-sensitive fruit, and low levels of ethylene can accelerate softening during storage. In this study, we used an ethylene absorbent (EA) and 1-methylcyclopropene (1-MCP) to minimize the detrimental impact of ethylene on the quality of peaches in modified atmosphere packaging (MAP), and analyzed fruit firmness, color change, anthocyanin content, and the expression patterns of cell wall metabolism-related genes and anthocyanin synthesis-related genes during storage. The results showed that ethylene in the MAP package decreased the firmness and total anthocyanin content of the peaches, while MAP combined with EA (MAP+EA) treatment effectively maintained the firmness of the peaches and counteracted the inhibition of anthocyanin accumulation in the peach skin by ethylene. In addition, the peaches treated with MAP+EA exhibited higher a* values, lower weight loss, and lower activities of cell-wall-modifying enzymes. Meanwhile, MAP+EA treatment also significantly increased the expression of color-related genes such as flavonoid 3′-hydroxylase gene (*F3′H*), dihydroflavonol 4-reductase (*DFR*), anthocyanidin synthase (*ANS*), and UDP-flavonoid 3-O-glucosyltransferase (*UFGT*). Furthermore, a good synergistic effect was observed between 1-MCP and EA in delaying softening and promoting coloring of peach fruit in the MAP package. The combination of 1-MCP and EA treatment may have the potential to alleviate softening and improve the color and quality of post-harvest fruit during storage.

## 1. Introduction

Peach (*Prunus persica* (L.) Batsch) is an important economic fruit crop in the Rosaceae family [1]. Honey peach is a type of peach fruit that is popular with consumers around the world due to its beautiful appearance, unique flavor, and nutritional value [2,3,4]. However, honey peach is extremely susceptible to deterioration during transport and storage, mainly characterized by water loss, rapid textural softening, and physical damage [5,6]. Excessive post-harvest softening of honeydew peach fruit results in short shelf life and high post-harvest losses, limiting the market value of the fruit [7]. In addition, as a climatic fruit, peaches usually ripen in summer with high temperatures during transport and sales. Therefore, how to improve the shelf-life quality of honey peach at high temperatures has become a major challenge [1]. In recent years, various chemical and physical techniques have been investigated to maintain the post-harvest quality of peach, including salicylic acid, 1-methylcyclopropene, cold storage, heat treatment, irradiation, controlled atmosphere, and modified atmosphere packaging (MAP) [8]. MAP is one of the most widely accepted methods of extending the shelf life of fruit, which can create a gaseous environment with low oxygen (O_2_) and high carbon dioxide (CO_2_) concentrations in the package [9]. MAP is commercially used to delay post-harvest quality loss of peach fruit during cold storage [10,11]. However, as a well-known climacteric fruit, peach may undergo anaerobic respiration and softening in MAP at high temperatures [4,12], due to the low-oxygen, high ethylene conditions and the increased respiration of fruit. Moreover, peaches usually mature in summer, with high temperatures during transportation and storage. Therefore, how to enhance the quality of peach fruit at room temperature has become a major challenge.

Ethylene is a gas produced by fruit that can adversely affect fruit nutrition, quality, and marketability during storage [13]. Many studies have confirmed that ethylene is closely associated with softening and senescence of fruit [14,15]. High levels of ethylene exposure would lead to premature ripening, increased spoilage and reduced shelf-life, resulting in significant post-harvest losses [16]. Loss of firmness in peaches was found to be very sensitive to the increased ethylene production [17]. Ethylene treatment resulted in rapid flesh softening by increasing the activity of cell-wall-degrading enzymes, including polygalacturonase (PG), pectin methylesterase (PME), cellulase (CEL), and β-galactosidase (β-GAL) [18,19]. In addition, our previous research found that ethylene could inhibit the accumulation of anthocyanins in peach skin after harvest [2]. Exogenous ethylene treatment significantly suppressed the expression of genes associated with anthocyanin biosynthesis, such as dihydroflavonol 4-reductase (*DFR*), anthocyanidin synthase (*ANS*), and UDP-glucose:flavonoid 3-O-glucosyltransferase (*UFGT*) genes [2]. Therefore, the accumulated ethylene levels in MAP may exacerbate softening and inhibit coloring of peaches. Minimizing the deleterious effects of ethylene in the package can effectively maintain fruit quality and retard ethylene-induced deterioration during storage [13]. Ethylene absorbent (EA) is the most widely used external ethylene scavenger, which shows good potential for inhibiting ethylene production and prolonging the post-harvest life of fruit [20,21]. On the other hand, ethylene inhibitors such as 1-methylcyclopropene (1-MCP) and aminoethoxyvinylglycine (AVG) play a negative role in modulating the ripening process of many climacteric fruits [22].

Although both MAP and ethylene control technologies have been extensively studied separately in a variety of fruits [23], there is little research on the detrimental effects of the ethylene gas in MAP on the anthocyanin synthesis and softening in honeydew peach at high temperatures. Therefore, in the present study, we conducted experiments to evaluate the combined effects of MAP and ethylene control (EA and 1-MCP) treatment on anthocyanin synthesis and softening of peach. The aim of this work was to investigate whether EA treatment could counteract the side-effect of ethylene in MAP on anthocyanin synthesis in post-harvest peach and have a synergistic effect on delaying softening [18]. The results could provide a convenient and feasible method of preserving honey peach during transport and storage.

## 2. Materials and Methods

### 2.1. Material and Treatments

Honey peach (*Prunus persica* (L.) Batsch cv. ‘Baifeng’ and ‘Xinchuanzhongdao’) fruits were obtained from a commercial orchard in Suqian City, which were bagged with double-layer fruit bags before harvesting. First, a total of 360 ‘Baifeng’ peaches were randomly divided into three groups for the following treatments: (1) control (Control), eight peaches were placed in polyethylene bags (0.02 mm, 60 × 40 cm) with eight punches (1 cm diameter each); (2) modified atmosphere packaging (MAP), eight peaches were placed in sealed polyethylene bags (high oxygen permeability, 0.02 mm, 60 × 40 cm); (3) modified atmosphere packaging combined EA (MAP+EA), eight peaches and 5 g KMnO_4_ were placed in sealed polyethylene bags. Next, a total of 720 ‘chuanzhongdao’ peaches were randomly divided into six groups for the following treatments: (1) control (Control), eight peaches were treated with air in a sealed plastic container for 24 h and then placed in polyethylene bags with eight punches; (2) MAP treatment (MAP), eight peaches were treated with air in a sealed plastic container for 24 h and then placed in sealed polyethylene bags; (3) MAP combined EA treatment (MAP+EA), eight peaches were treated with air in a sealed plastic container for 24 h and then placed in sealed polyethylene bags with 5 g KMnO_4_; (4) 1-MCP treatment (1-MCP), eight peaches were treated with 1.5 μL·L^−1^ 1-MCP for 24 h in a sealed plastic container and then placed in polyethylene bags with eight punches; (5) 1-MCP combined MAP treatment (MAP+1-MCP), eight peaches were treated with 1.5 μL·L^−1^ 1-MCP for 24 h in a sealed plastic container and then placed in sealed polyethylene bags; (6) 1-MCP combined MAP and EA treatment (MAP+EA+1-MCP), eight peaches were treated with 1.5 μL·L^−1^ 1-MCP for 24 h in a sealed plastic container and then placed in sealed polyethylene bags with 5 g KMnO_4_.

Experiments were performed in triplicate, and the treatments were carried out at room temperature (22 ± 2 °C). Three packages were randomly selected after 0, 2, 4, 6, and 8 d for physical and chemical analysis.

### 2.2. Determination of the Weight Loss and Firmness of Peach

The weight loss of every treatment was measured by weighing the peach at the beginning and end of the monitoring period [24].

$$Weight\;loss\;(\%)=\frac{W_{o}-W_{1}}{W_{o}}\times 100$$

where W_o_ and W_1_ denote the weight at the beginning and end of the monitoring period, respectively.

A sclerometer (TP-GY-4, Zhejiang Top Instrument Co., Ltd., Hangzhou, China) with a 7.9 mm probe was used to determine the firmness of 8 peaches [25]. Firmness was expressed as the maximum force (N) obtained.

### 2.3. Determination of the Respiratory Rate and Ethylene Production of Peach

Four fruits per group were kept at the same temperature for 4 h in a 4.5 L airtight plastic container. A 10 mL gas sample was taken from the containers and analyzed for CO_2_ and ethylene content. The composition of the gases was determined by gas chromatography (GC-7820, Agilent Technologies Inc., Santa Clara, CA, USA ) as described by our previous study with at least three replicates for each group [2]. The CO_2_ and ethylene content of the gas samples were compared to a standard CO_2_ and ethylene mixture of known concentrations (1000 ppm CO_2_ and 5 ppm ethylene) based on peak area. The respiration rate and ethylene production rate of peach were expressed as CO_2_ (mg) and ethylene (C_2_H_4_) (μL) produced per hour per kilogram (FW), respectively [26].

### 2.4. Determination of the Gaseous Composition in the Package

The gaseous composition (O_2_ and CO_2_ concentration) of different peach packages was monitored at periodic intervals with a gas analyzer (CheckMate 3, PBI Dansensor, Ringsted, Denmark) [27]. The ethylene concentration in the package was measured by gas chromatography according to our previous study [2]. A quantity of 5 mL of headspace gas in the package was sampled and injected into a gas chromatograph (GC-7820). The ethylene concentration in the package was compared to a standard ethylene of known concentration (5 ppm ethylene) based on peak area. Three independent experiments were performed for each sample.

### 2.5. Measurement of Enzyme Activities of Cell-Wall-Modifying Enzymes

PG and PME activities were assessed using the method described by Deng et al. (2015) with slight modifications [28]. Briefly, 2.0 g of frozen peach flesh was homogenized with 4.0 mL of an extraction solution containing 6.8% NaCl, 1% PVP and 0.6% EDTA. The resulting homogenate was then centrifuged at 11,000× *g* at 4 °C for 20 min, after which the supernatant was collected and used for the enzymatic assay.

Determination of PG enzyme activity: 0.5 ml of acetic acid-sodium acetate buffer solution (pH 4.6, 50 mM), 0.4 ml of a 10 g/L polygalacturonic acid solution, and 0.1 mL of an enzyme extract were mixed together. The reaction system was then incubated at 37 °C for one hour before 1.0 mL of 3,5-dinitrosalicylic acid reagent was quickly added. The reaction system was then boiled for 5 min, cooled, and adjusted to 10 mL with distilled water. The absorbance at 540 nm of the reaction mixture was measured [28]. The PG enzyme activity was expressed as the mass of polygalacturonic acid converted into galacturonic acid at 37 °C, with activity expressed in g kg^−1^ h^−1^ [18].

Determination of PME enzyme activity: 0.2 ml of the enzyme extraction was mixed with 0.1 ml of 0.01% (*w/v*) bromophenol blue indicator and 1 ml of 1% pectin solution (pH 7.5) was added as the substrate. The changes in the reaction solution’s absorbance at 620 nm were then recorded. PME activity was defined as the amount of enzyme required to liberate methyl ester; PME activity is expressed in mmol kg^−1^ min^−1^ [29].

β-Galactosidase (β-GAL) and cellulase (CEL) activity was measured using a previously described method [29]. A frozen peach sample (2 g) was ground with 8 mL of a 40 mM NaAc solution (pH 5.5), 2 mL of a 0.2 M NaCl solution, and 10 g/L polyvinyl pyrrolidone. The homogenate was then centrifuged at 11,000× *g* at 4 °C for 20 min. The resulting supernatant was collected for use in the assay of β-GAL and CEL activities.

Determination of β-galactosidase (β-GAL) enzyme activity: 0.1 mL of the enzyme extract solution was added to 0.5 mL of a sodium acetate (NaAc) solution (40 mM, pH 5.0) and 0.4 mL of a p-nitrophenyl-β-D-galactopyranoside solution (6 mM), and the mixture was incubated for 1 h at 40 °C. The reaction was then terminated by the addition of 1.0 mL of 0.5 mol/L Na_2_CO_3_. β-GAL enzyme activity was determined at 400 nm using p-nitrophenol as a standard. β-Gal activity was defined as the quantity of enzyme needed to generate 1 mmol of p-nitrophenol per hour; β-GAL activity is expressed in mmol kg^−1^ h^−1^ [18].

Determination of CEL enzyme activity: 1.0 mL of enzyme extract was mixed with 2.0 mL of 10 g/L sodium carboxymethyl cellulose. The mixture was incubated at 37 °C for 1 h, after which 1.5 mL of DNS reagent was quickly added. The mixture was then boiled for 5 min, cooled, and the reaction system adjusted to 10 mL with distilled water. The cellulase enzyme activity was measured at 540 nm, calculated against glucose as the standard [29], and expressed as g kg^−1^ h^−1^ [18].

### 2.6. Determination of the Peel Color of Peach

The peel color was determined for eight different peaches. Four equatorial regions of each peach were measured using a colorimeter (CR-400, Konica Minolta, Tokyo, Japan). The chromatic coordinates of the peaches were recorded. The a* coordinate shows green–red values ranging from −90 to 90 [2].

### 2.7. Determination of the Contents of Anthocyanin

One gram of frozen peel tissue was added to 4.0 ml of a 0.1% (*v*/*v*) methanol solution of HCl for grinding and extracting anthocyanins. The procedure was performed at 4 °C in darkness for 12 h, and then centrifuged at 12,000× *g* for 20 min. Then, 1 mL of extract was added to 4 mL of NaAC (pH 4.5) and 4 mL KCl (pH 1.0), respectively. The absorbance values were determined at 510 and 700 nm using the spectrophotometer. The total anthocyanin content was measured using the pH difference method [18]. The absorbance data were converted into total anthocyanin content (mg/kg) using the molar extinction coefficient of cyanidin-3-O-glucoside [26,900 L/(cm·mol)].

### 2.8. RNA Isolation and Quantitative Real-Time PCR Analysis

Total RNA was extracted from frozen peach flesh and peel tissues using the Trizol Plant Kit (TransGen Biotech, Beijing, China), following the manufacturer’s protocol. Real-time quantitative PCR (qPCR) was performed with TaKaRa SYBR Premix Ex Taq™ II (Takara Biomedical Technology Co., Ltd., Dalian, China) using a CFX96 instrument (Bio-Rad, Hercules, CA, USA). *TEF2* was used as the reference gene for normalizing all target gene expressions, including polygalacturonase (*PG*), pectin methylesterase (*PME*), β-galactosidase (*β-GAL*), pectate lyase (*PLY*), phenylalanine ammonia lyase (*PAL*), chalcone synthase (*CHS*), chalcone isomerase (*CHI*), flavanone-3-hydroxylase (*F3H*), flavonoid 3′-hydroxylase (*F3′H*), dihydroflavonol 4-reductase (*DFR*), anthocyanidin synthase (*ANS*), and UDP-glucose-flavonoid glycosyltransferase (*UFGT*) [19]. The relative expression levels of the target genes were calculated using the 2^−ΔΔCT^ method [30].The primer sequences are shown in detail in Table S1.

### 2.9. Statistical Analysis

Each analysis was carried out in triplicate. Data analysis was performed using SPSS 22.0 software. A one-way analysis of variance was used to assess the data with post hoc comparisons using Duncan’s multiple range tests. The results were expressed as the mean ± standard deviation, and *p* < 0.05 was considered statistically significant and indicated with a different letter.

## 3. Results

### 3.1. Visual Appearance Changes of the Peaches

**Figure 1:**
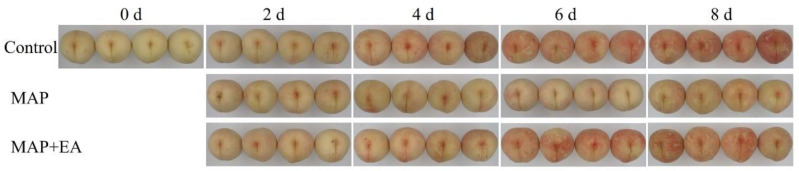
Effect of MAP and MAP+EA treatments on the visual appearance of peaches during storage at 25 °C for 8 days.

The color of the fruit peel is an important factor for evaluating the commercial value of peaches. As can be seen in Figure 1, the peaches were relatively white at 0 days due to being bagged before harvest. With the extension of storage time, the fruit gradually turned red on the second day, reaching their reddest color on the sixth and eighth days of storage. Compared with the control group, the peaches in the MAP treatment group colored later and exhibited lighter coloration. MAP treatment had an inhibitory effect on the coloring of the peaches. Meanwhile, the MAP+EA treatment reduced the inhibitory effect of the MAP treatment on the coloring of the peaches. The degree of pigmentation of the MAP+EA-treated peaches in the later stage of storage was almost the same as that of the control group.

### 3.2. Changes in Weight Loss and Firmness of Peach During Storage

**Figure 2:**
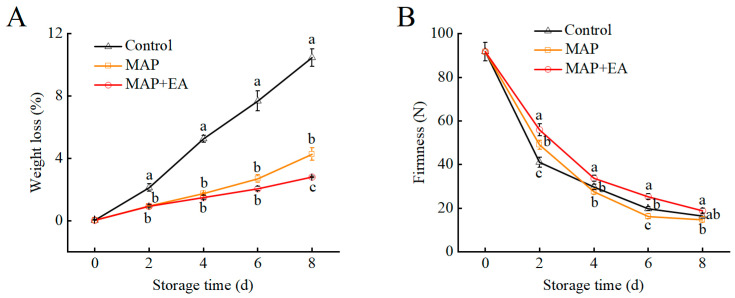
Effect of MAP and MAP+EA treatments on weight loss (**A**) and firmness (**B**) of peach fruit during storage at 25 °C for 8 days. The differences among the three treatments, indicated with a different letter vertically, were significant at *p* < 0.05 within the same day.

As shown in Figure 2A, the weight loss of peach for each treatment gradually increased during storage. The MAP and MAP+EA treatments effectively inhibited the increase in the weight loss rate of the peaches. It is also worth noting that the weight loss of the control treatment was more than 7% on the 6th day. However, the weight loss of peach fruit in the MAP and MAP+EA treatments was much lower—below the acceptable limit of 5%. Softening is one of the main physiological characteristics that affect the shelf life of peaches. The firmness of the peach fruit rapidly decreased and then slowly declined in all treatments during storage (Figure 2B). On day 2, the fruits in the MAP and MAP+EA groups was firmer than those in the control group. EA treatment inside the package has a positive effect on peach fruit firmness. The hardness of peach fruits in the MAP+EA treatment group was significantly higher than that in the control group and the MAP treatment group in the first 6 days of storage (*p* < 0.05). On the second day of storage, the hardness of the MAP+EA treated fruits was 1.36 times and 1.14 times that of the control group and the MAP treatment group, respectively. However, there was no significant difference in firmness between the control and the MAP-treated peaches during the later stage of storage.

### 3.3. Respiration Rate and Ethylene Production Rate of Peach During Storage

**Figure 3:**
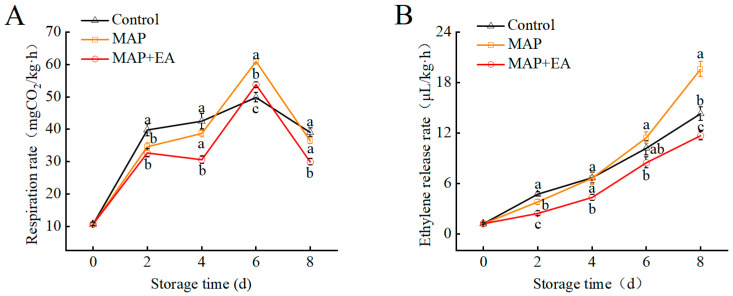
Effect of MAP and MAP+EA treatments on the respiration rate (**A**) and ethylene production rate (**B**) of peach fruit during storage at 25 °C for 8 days. The differences among the three treatments, indicated with a different letter vertically, were significant at *p* < 0.05 within the same day.

The respiratory rate of the peaches increased gradually during the first six days of storage and decreased on the eighth day (Figure 3A). Compared with the control group, the MAP and MAP+EA treatments effectively inhibited the increase in the respiration rate of the peaches on the second day of storage (*p* < 0.05). However, on the sixth day of storage, the respiration rate of the fruit in the MAP and MAP+EA groups increased sharply. Throughout the storage period, the respiration rate of peaches in the MAP+EA treatment group was significantly lower than that in the MAP treatment group (*p* < 0.05). The ethylene production rate of the peach fruits in all the three groups gradually increased during storage (Figure 3B). Compared with the control group, MAP+EA treatment significantly inhibited the increase in ethylene production rate during storage (*p* < 0.05), except on day 6. However, the ethylene production rate of the MAP treatment group was lower than that of the control group only on day 2 (*p* < 0.05), and higher on day 6. Furthermore, the ethylene production rate of the MAP treatment group was significantly higher than that in the MAP+EA treatment group or control group throughout the storage period (*p* < 0.05).

### 3.4. Gas Composition Inside the Packages

**Figure 4:**
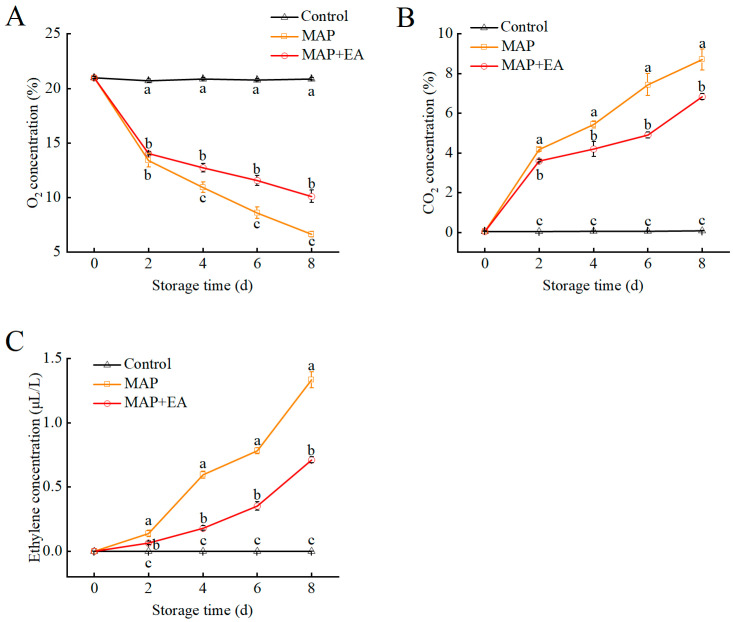
Effect of MAP and MAP+EA treatments on the O_2_ concentration (**A**), CO_2_ concentration (**B**), and ethylene concentration (**C**) inside the different packages during storage at 25 °C for 8 days. The differences among the three treatments, indicated with a different letter vertically, were significant at *p* < 0.05 within the same day.

The levels of O_2_, CO_2_, and C_2_H_4_ detected in the headspace of all the packages during storage are shown in Figure 4. The control group was kept in plastic bags with several holes and directly exposed to air without MAP treatment; therefore, the O_2_, CO_2_, and C_2_H_4_ contents were similar to those found in the air. Compared with the control group, the gas composition inside the MAP and MAP+EA packages changed rapidly, and we observed a decrease in O_2_ concentration as well as an increase in CO_2_ and C_2_H_4_ concentration during storage. The O_2_ content in both the MAP treatment group and the MAP+EA treatment group significantly decreased after the second day of storage (Figure 4A), while the O_2_ content in the MAP+EA treatment group was significantly higher than that in the MAP treatment group after the fourth day of storage (*p* < 0.05). The CO_2_ content in the MAP+EA treatment group was significantly lower than that in the MAP treatment group throughout the storage period (*p* < 0.05) (Figure 4B). We observed that the ethylene content in the MAP treatment group was significantly higher than that in the MAP+EA treatment group or control group during storage (Figure 4C). On the eighth day of storage, the ethylene content in the MAP treatment group was 1.88 times higher than that in the MAP+EA group (Figure 4C).

### 3.5. Changes in the Activity of Cell-Wall-Modifying Enzymes of Peaches During Storage

**Figure 5:**
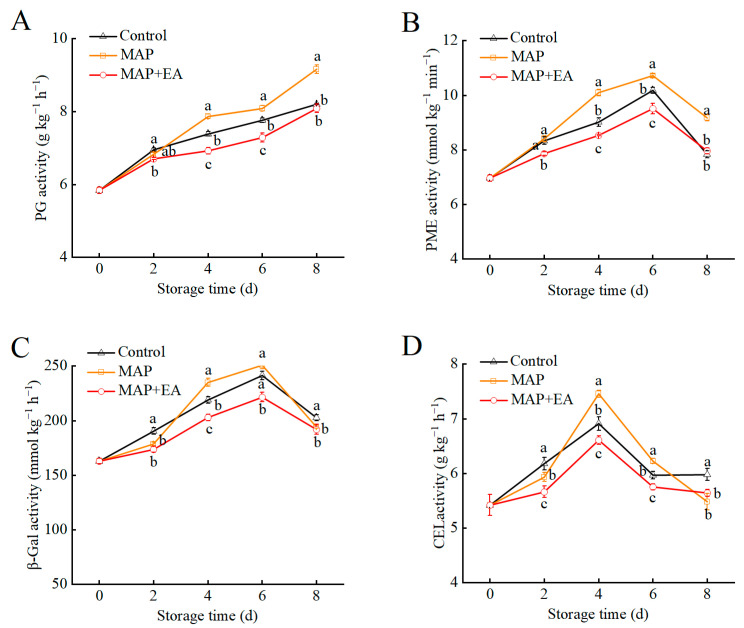
The effect of MAP and MAP+EA treatments on the PG (**A**), PME (**B**), β -Gal (**C**), and CEL (**D**) activities of peach fruit during storage at 25 °C for 8 days. The differences among the three treatments, indicated with a different letter vertically, were significant at *p* < 0.05 within the same day.

The impacts of the MAP and MAP+EA treatments on the enzyme activities of the cell-wall-degrading enzymes in peach fruit are shown in Figure 5. The PG activity in peaches steadily increased with storage time in all three treatments (Figure 5A). With the extension of the shelf life, the PG activity in the MAP-treated fruit was significantly higher than that in the control after day 4 (*p* < 0.05). However, the MAP+EA treatment delayed the increase in PG activity in peaches up to day 6. The activities of the PME and β-Gal enzymes showed a similar trend in peaches during storage (Figure 5B,C). The activities of PME and β-Gal increased during the initial storage period, reaching maximum activity on the sixth day, followed by a sharp decline. Generally, the PME and β-Gal enzyme activities in the MAP+EA-treated fruits were lower than in the control group on days 2–6 during storage. Compared to control fruits, the PME and β-gal activities were markedly higher in MAP fruits on day 4. The CEL activity in MAP+EA-treated fruit was consistently lower than that in the control and MAP groups during storage, suggesting CEL activity was inhibited in MAP+EA-treated peaches (Figure 5D).

### 3.6. Changes in the Expression of Genes Associated with Flesh Softening

**Figure 6:**
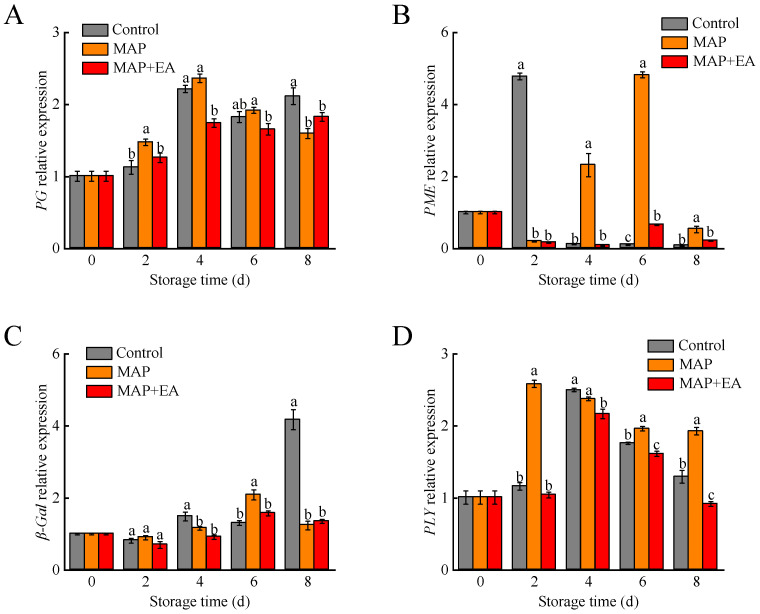
The effect of MAP and MAP+EA treatments on the gene expressions of *PG* (**A**), *PME* (**B**), *β-Gal* (**C**), and *PLY* (**D**) of peach fruit during storage at 25 °C for 8 days. The differences among the three treatments, indicated with a different letter vertically, were significant at *p* < 0.05 within the same day.

The expression of several genes involved in cell wall degradation, including *PG*, *PME*, *β-Gal*, and *PLY*, was examined using quantitative real-time PCR (Figure 6). *PG* expression increased significantly in all three samples after the second day of storage (Figure 6A). However, PpPG expression in the MAP+EA treatment group was lower than in the control and MAP groups on the fourth day (*p* < 0.05). The expression level of the *PME* gene in the control peaches increased sharply on the second day, reaching a value significantly higher than that in the MAP and MAP+EA treatment groups (Figure 6B). Moreover, the abundance of *PME* transcripts in the MAP samples increased significantly on the fourth day and reached the highest peak value on the sixth day, which was approximately 7.29-fold higher than that in the MAP+EA group on the sixth day. The β-*Gal* gene expression in the MAP+EA treatment group was significantly lower than in the control group on days 4 and 8 (Figure 6C). As shown in Figure 6D, the MAP+EA treatment significantly down-regulated the expression level of the *PLY* genes, which were lower than those of the control and MAP treatment groups on days 4, 6, and 8 (*p* < 0.05).

### 3.7. Changes in the Color Difference Value and Total Anthocyanin Contents of Peach Peel

**Figure 7:**
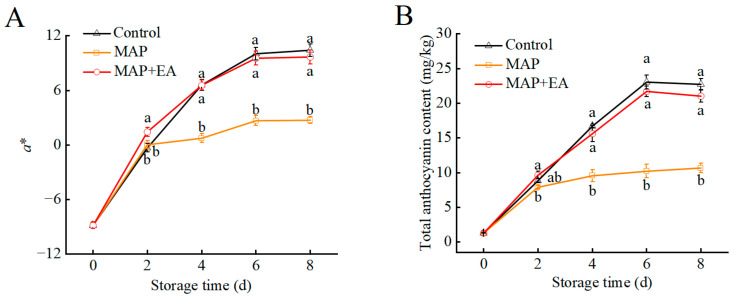
Effect of MAP and MAP+EA treatments on the a* value (**A**) and total anthocyanin content (**B**) of peach peel during storage at 25 °C for 8 days. The differences among the three treatments, indicated with a different letter vertically, were significant at *p* < 0.05 within the same day.

As shown in Figure 7A and Figure S1, the color difference values (a*, b*, and L*) of the peach peel obviously changed during storage. The a* value and total anthocyanin content of peach peel increased gradually during storage in all the three groups (Figure 7) and showed a similar change trend. Compared with the control group, MAP treatment suppressed the increase in the a* value and total anthocyanin content from days 4 to day 8. However, MAP+EA reduced the inhibitory effect of sealed packaging on increase in the a* value and total anthocyanin content during the later stages of storage (Figure 7A,B). On the eighth day of storage, the anthocyanin content of peach peel in the Control group and MAP+EA groups was 2.13 and 1.97 times higher than in the MAP group, respectively (Figure 7B). Therefore, the peel of fruit in the Control and MAP+EA groups appeared redder than that of fruit in the MAP group during the later storage period. This suggests that the ethylene absorbent used in the MAP packaging had a positive effect on total anthocyanin content during storage.

### 3.8. Changes in the Expression of Genes Associated with Anthocyanin Metabolism

**Figure 8:**
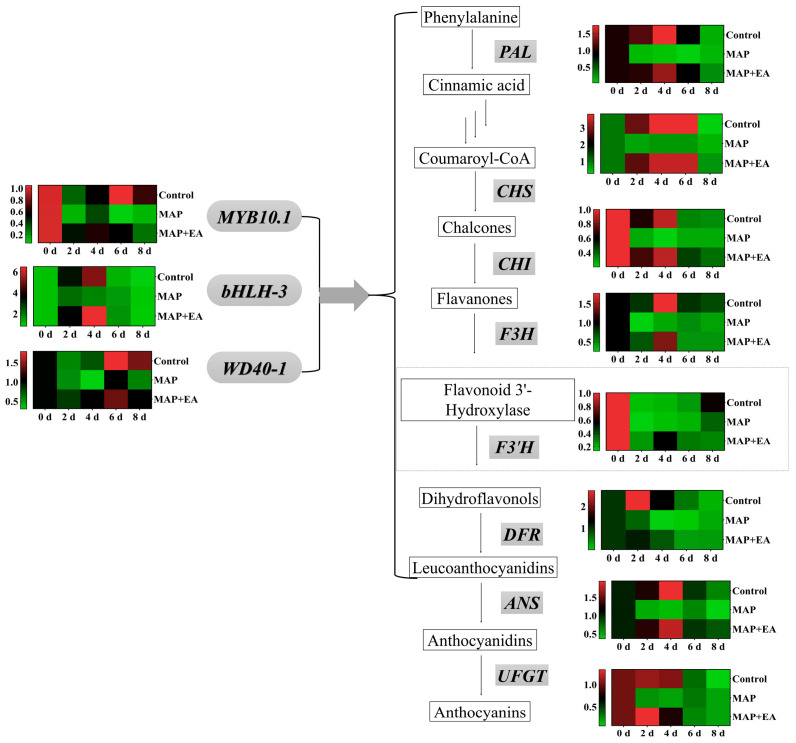
Effect of MAP and MAP+EA treatments on the expressions of structural genes and transcription factors associated with anthocyanin biosynthesis in peach peel during storage at 25 °C for 8 days.

The effect of MAP and MAP+EA treatments on the expression pattern of genes involved in anthocyanin metabolism are presented in Figure 8. The relative expressions of most structural genes associated with anthocyanins biosynthesis of the control group remained at a relatively high level during the early stage of storage and decreased significantly during the later stage of storage. The highest relative expression of the *DFR* and *UFGT* gene was observed on the second day in the control, while the genes coding *PAL, CHS, CHI, F3H*, and *ANS* reached their relative highest expression on day 4. In general, the MAP treatment significantly reduced the expression of the anthocyanin biosynthesis genes during almost the entire storage period, except on day 8. Compared to the MAP treatment group, the MAP+EA treatment significantly increased the expression levels of *PAL*, *CHS*, *CHI*, *F3H*, *DFR*, *ANS*, and *UFGT* genes in peach peels in the early part of the storage period. On the second day, the expression levels of *DFR* and *UFGT* genes in the MAP+EA treatment group were 1.63 times and 5.84 times that of the MAP treatment group, respectively. Furthermore, the expression level of the *UFGT* gene in the MAP+EA group was significantly higher than that in the Control group on day 2.

We also investigated the effects of the MAP and MAP+EA treatments on the expression of genes which encoded for the transcription factors involved in the anthocyanin biosynthesis of peach (Figure 8). The highest relative expression of the *MYB10.1* and *WD40-1* transcription factors in the control group was observed on day 6, while the highest expression of the *bHLH3* transcription factor was found on day 4. MAP treatment also reduced the expression of *MYB10.1*, *bHLH3*, and *WD40-1* genes, which was similar to the effects of MAP on the expression of most structural genes associated with anthocyanins biosynthesis. Compared with the MAP treatment group, the MAP+EA treatment significantly increased the expression of *MYB10.1*, *bHLH3*, and *WD40-1* genes. On the 4th day of storage, the expression level of *bHLH-3* in the MAP+EA treatment group was 1.25 times and 3.57 times that of the Control group and the MAP treatment group, respectively.

### 3.9. Effects of 1-MCP Combined EA with Treatment on the Anthocyanin Content and Firmness of Peach

**Figure 9:**
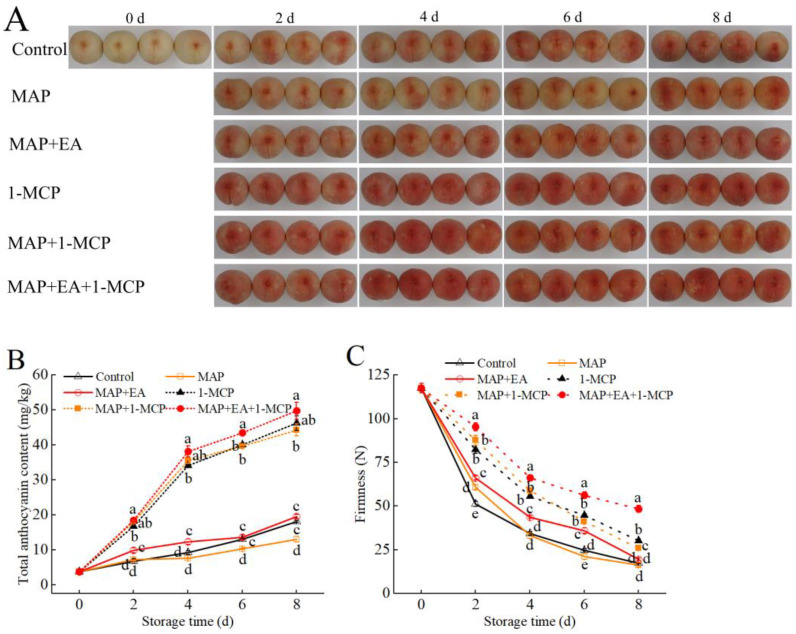
Effects of 1-MCP combined with EA treatment on the appearance (**A**), the firmness (**B**), and total anthocyanin content (**C**) of peach during storage at 25 °C for 8 days. The differences among the three treatments, indicated with a different letter vertically, were significant at *p* < 0.05 within the same day.

The effects of EA combined with 1-MCP treatment on the coloration of peach are shown in Figure 9A. Compared with the control group, almost all of the treatments (except MAP) accelerated the pigmentation process. Furthermore, color development in the MAP+EA-treated fruit occurred earlier than in the control on the second day, and the extent of redness was similar to that in the control group at the end of storage. Consistent with the visual changes in color, significant variations in anthocyanin content were observed in all groups (Figure 9B). The lowest anthocyanin content was observed in the MAP treatment at the end of storage, exhibiting a similar trend to that shown in Figure 7B. It is worth noting that the EA and 1-MCP treatments also promoted the accumulation of total anthocyanins; however, the anthocyanin content of 1-MCP-treated fruit increased earlier and to a greater extent than that of the MAP+EA groups. Compared with the control group, the 1-MCP treatment significantly improved anthocyanin biosynthesis, reaching 2.55 times that of the control group by day 8. Fruit treated with MAP+EA+1-MCP had the highest total anthocyanin content compared with the other treatment groups throughout the storage period, reaching 3.82 times that of the MAP group on day 8. Similarly to the previous study, the hardness of peach fruit in the control group and the MAP treatment group decreased significantly during storage (Figure 9C). Compared with the control, MAP and MAP+EA groups, the hardness of peach fruit in the 1-MCP treatment groups was better maintained, while the hardness of peach fruit treated with MAP+EA+1-MCP remained the highest throughout the shelf life.

### 3.10. Effect of 1-MCP Combined with EA Treatment on the Expression of Genes Associated with Anthocyanin Metabolism

**Figure 10:**
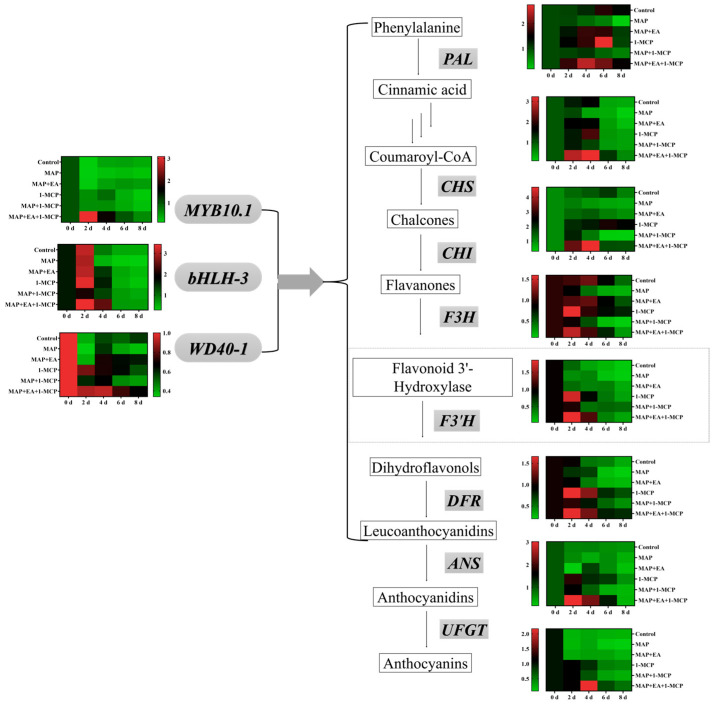
Effect of 1-MCP combined with EA treatment on the expression levels of structural genes and transcription factors associated with anthocyanin biosynthesis in peach peel during storage at 25 °C for 8 days.

Similarly with the color change, the relative expression levels of most genes associated with anthocyanin biosynthesis increased significantly in the early stage of storage (Figure 10). Compared with the MAP treatment group, the MAP+EA treatment also increased the expression levels of *PAL*, *CHS*, *F3H*, and *DFR* genes on day 2. Compared with the control and EA treatment, the 1-MCP treatment significantly increased the expression of most anthocyanin synthesis-related genes on day 2 (*p* < 0.05), such as *CHS, F3H*, *F3′H*, *DFR, ANS*, and *UFGT*. The expression of all the genes in the MAP+EA+1-MCP treatment group was almost at the highest level throughout the storage period on day 2. On day 4 of storage, the expression levels of the *PAL, CHS, CHI, F3′H*, and *UFGT* genes in the MAP+EA+1-MCP treatment group were 2.08, 1.95, 3.03, 5.63, and 7.76 times those in the control group, respectively. On the 8th day of storage, the expression of most genes related to anthocyanin synthesis decreased significantly compared with that in the early stage of storage. However, the expression levels of the *PAL, CHS, CHI, F3H, F3′H, DFR, UFGT, MYB10.1, bHLH-3*, and *WD40-1* genes in the MAP+EA+1-MCP treatment group were significantly higher than those in the control group (*p* < 0.05).

## 4. Discussion

The shelf life of peaches can be considerably affected by many extrinsic factors, such as temperature, humidity, and atmosphere [31], which lead to various biochemical and physiological changes in peaches during storage. Nowadays, MAP is a novel handling method commonly used to inhibit post-harvest weight loss and delay softening, extending the shelf life of fresh peaches [31]. Compared to the control, MAP treatments could retard the reduction in titratable acidity and total soluble solid, and decrease the development of softness of peach during storage at 2 °C [32]. However, Mahajan et al. found that LDPE packaging caused a very high level of CO_2_ accumulation in the package, which led to formation of fermenting odor and decay of peach ambient sale conditions [33]. New integrated structure packaging had a significant effect on extending the shelf life of peach, which has high water vapor and high oxygen permeability [27]. In our previous research, one type of film with high oxygen permeability was used for MAP treatment which could effectively maintain the hardness of the peaches and extend their shelf life [34]. However, compared to control (perforated package), we found that MAP treatment only delayed the decline in peach firmness on the second day, and accelerated the softening of peach on the sixth day in this research, which may be due to the vigorous physiological metabolism of the peaches and gas environment inside the package [34]. For climacteric fruits, such as peach, apple, banana, and so on, ethylene is a key hormone associated with ripening and senescence process [35]. The respiration rate and senescence of many fruits have also been promoted by ethylene [36]. It has been reported that ethylene gas can change the chemical and physical stability of plants at very low concentration in the range of 10–100 nL·L^−1^ [13,37]. Therefore, the high concentration of ethylene in the headspace of peach packaging in this study may be the main cause of spoilage [35], which was up to approximately 1.0 μL·L^−1^.

Controlling the ethylene content in the package using effective and safe approaches is key to extending the post-harvest shelf life of peach. Many ethylene-controlled technologies, including ethylene removal and ethylene synthesis suppression technologies, have been used to extend the shelf life of fruit [1,13,38,39]. Cheng et al. found that 1-MCP and EA significantly delayed ethylene release, reduced the respiration rate, and prolonged the shelf life of peaches during E-commerce logistics [1]. Li et al. also showed that a metal-organic framework could effectively absorb the ethylene gas in storage packaging and delay kiwi fruit ripening [39]. Compared to the MAP treatment, EA treatment inside the package significantly reduced the ethylene content in the headspace of the package and had a positive effect on peach fruit firmness. This finding supports previous studies where EA treatments for blueberry [14] and banana [40] consistently preserved fruit firmness [41]. The degradation of cell wall components is the main reason for the softening of fruits, and many cell-wall-modifying enzymes play important roles during this senescence process, such as polygalacturonase (PG), pectin methylesterase (PME), β-galactosidase (β-gal), and cellulase (CEL) [18,42]. In this study, a continuous increase in PG was observed, and the activities of PME and β-Gal peaked on day 6 (Figure 5), while MAP+EA treatment significantly inhibited the activities of PG, PME, and β-Gal (*p* < 0.05). Similarly, markedly suppressed increases in PG, PME, and β-Gal enzymes activities were noted in 1-MCP-treated flat peach [7]. A previous study also indicated that the application of hot air and 1-MCP cloud suppress PG and PME activities, thereby retarding nectarine softening [18]. In addition to the cell-wall-modifying enzymes, the expression levels of *PG*, *PME*, *β-Gal*, and *PLY* genes were also reduced in the MAP+EA treatment group at some time points during storage. Similarly, Shi et al. (2019)reported that the inhibited expression of cell-wall-modifying enzymes genes (*β-Gal*, *PG*, and *PME*) could suppress the disassembly of cell wall polysaccharides [43], which has been linked to grapefruit softening.

The peel color of peach in this research is white when it is just harvested, and it turns red as the storage time increases. The red color of the skin is mainly due to the accumulation of anthocyanins in the peel; cyanidin-3-glucoside is the main anthocyanin monomer in the peel [2]. Ethylene is a crucial phytohormone that plays a pivotal role in regulating fruit ripening traits like color and firmness [22]. In many fruits, ethylene enhances coloration and anthocyanin biosynthesis, such as in apples [44], mango [45], and plum [46]. However, ethylene also had a negative effect on anthocyanin biosynthesis in several species, including pear [47] and peach [2]. Our previous research found that ethylene will inhibit the skin red coloration in post-harvest peach [2]. Similarly, the present study showed that the ethylene content in the MAP treatment was relatively higher, and peaches in the MAP treatment group had lighter reddish color, which was reflected by the significantly lower a* value and anthocyanin content. Furthermore, we found that MAP+EA treatment effectively reduced the ethylene content in the package, and counteracted ethylene inhibition of anthocyanin accumulation in peach skin. Similarly, a previous study showed that the amount of anthocyanin in the ‘Silver Queen’ nectarine significantly increased with increase in storage, and the highest anthocyanin content was found in fruits packed with silica gel and KMnO_4_ [48]. However, Zheng et al. found that 1-MCP+EA treatment reduced anthocyanins accumulation and inhibited the red coloration in post-harvest nectarines [20]. Importantly, the effect of ethylene on anthocyanin accumulation in fruit is inconsistent, which may be due to differences in the type of fruit and the ripening stage.

The ethylene receptor inhibitor 1-MCP has similar effects on maintaining higher firmness and the nutritional value of peach [49]. Therefore, in the present study, the effects of combined 1-MCP and EA treatment on anthocyanin biosynthesis and firmness in honey peaches were investigated. Compared to EA treatment, 1-MCP is more effective in maintaining fruit firmness and promoting fruit coloring—the highest anthocyanin content and firmness were found in the 1-MCP+EA-treated peaches in this work. Similarly, our previous research found that 1-MCP treatment had a strengthening effect on coloration, and maintained the firmness of peach [2]. In order to gain insight into the mechanism underlying the positive effect of EA and 1-MCP on anthocyanin biosynthesis in peach skin, the expressions of the genes associated with anthocyanin biosynthesis were studied. In the present study, we found that EA treatment can effectively enhance the expression of multiple genes related to anthocyanin synthesis in two varieties, while the expression levels of all the genes in the 1-MCP-treated peach is higher than the EA group. Moreover, the highest levels of anthocyanin metabolism gene transcripts were found in EA+1-MCP-treated peaches, which is consistent with the anthocyanin content and fruit phenotype. However, the previous researches showed that 1-MCP treatment reduced the anthocyanin accumulation of nectarines [18] and plum [46]. These results showed that the effect of 1-MCP on anthocyanin accumulation in different fruits is inconsistent, suggesting that the metabolism of anthocyanin is complex.

## 5. Conclusions

In summary, we found that low concentration ethylene gas (approximately 0.1 μL·L^−1^) in the package caused flesh softening and decreased total anthocyanin accumulation in peach skin during storage by enhancing the activities of cell-wall-modifying enzymes and down-regulating the expression of many genes associated with anthocyanin biosynthesis, respectively. EA treatment can effectively reduce the ethylene content inside the package and has a positive effect on peach fruit firmness and coloration. The combination of 1-MCP and EA treatment not only delayed the softening but also promoted peach coloring in the MAP; the anthocyanin content was 3.82 times that of the MAP group. These results suggest that the synergistic application of EA and 1-MCP is a promising strategy for improving the quality of peaches during transportation and shelf life. Further studies should focus on elucidating the molecular mechanisms of the different effects of ethylene on anthocyanin synthesis in different fruits.

## Data Availability

The original contributions presented in this study are included in the article/Supplementary Materials. Further inquiries can be directed to the corresponding authors.

## Supplementary Materials

The following supporting information can be downloaded at: https://www.mdpi.com/article/10.3390/foods14142472/s1, Figure S1: Effect of MAP and MAP+EA treatments on the *L** value (A) and *b** value (B) of peach peel during storage at 25 °C for 8 days. Table S1: RT-qPCR primer sequences used in this study.

## References

[1] Cheng C., Liang X., Wei W., Zhang N., Yao G., Yan R. (2024). Enhanced shelf life quality of peaches (*Prunus persica* L.) using ethylene manipulating active packaging in e-commerce logistics. Sci. Hortic..

[2] Zhang Y., Ling J., Zhou H., Tian M.y., Huang W., Luo S., Hu H., Li P. (2022). 1-Methylcyclopropene counteracts ethylene inhibition of anthocyanin accumulation in peach skin after harvest. Postharvest Biol. Technol..

[3] Wang X., Fu D., Fruk G., Chen E., Zhang X. (2018). Improving quality control and transparency in honey peach export chain by a multi-sensors-managed traceability system. Food Control.

[4] Leng P., Hu H., Cui A., Tang H., Liu Y. (2021). HS-GC-IMS with PCA to analyze volatile flavor compounds of honey peach packaged with different preservation methods during storage. LWT-Food Sci. Technol..

[5] Hayama H., Tatsuki M., Ito A., Kashimura Y. (2006). Ethylene and fruit softening in the stony hard mutation in peach. Postharvest Biol. Technol..

[6] Qian J., Zhao Y., Shi Y., Chen K. (2022). Transcriptome analysis of peach fruit under 1-MCP treatment provides insights into regulation network in melting peach softening. Food Qual. Saf..

[7] Zheng Y., Jia X., Duan L., Li X., Zhao Z. (2023). Synergistic effects of 1-MCP fumigation and ε-Poly-L-Lysine treatments on delaying softening and enhancing disease resistance of flat peach fruit. Foods.

[8] Sortino G., Saletta F., Puccio S., Scuderi D., Allegra A., Inglese P., Farina V. (2020). Extending the shelf life of white peach fruit with 1-methylcyclopropene and aloe arborescens edible coating. Agriculture.

[9] Zhao Q., Jin M., Guo L., Pei H., Nan Y., Rao J. (2020). Modified atmosphere packaging and 1-methylcyclopropene alleviate chilling injury of ‘Youhou’ sweet persimmon during cold storage. Food Packag. Shelf Life.

[10] Özkaya O., Yildirim D., Dündar Ö., Tükel S.S. (2016). Effects of 1-methylcyclopropene (1-MCP) and modified atmosphere packaging on postharvest storage quality of nectarine fruit. Sci. Hortic..

[11] Akbudak B., Eris A. (2004). Physical and chemical changes in peaches and nectarines during the modified atmosphere storage. Food Control.

[12] Zhou H., Ye Z., Su M. (2018). Effects of MAP treatment on aroma compounds and enzyme activities in flat peach during storage and shelf life. HortScience.

[13] Sadeghi K., Lee Y., Seo J. (2019). Ethylene Scavenging Systems in Packaging of Fresh Produce: A Review. Food Rev. Int..

[14] Wang S., Zhou Q., Zhou X., Wei B., Ji S. (2018). The effect of ethylene absorbent treatment on the softening of blueberry fruit. Food Chem..

[15] Chen H., Lai X., Wang L., Li X., Chen W., Zhu X., Song Z. (2022). Ethylene response factor MaERF012 modulates fruit ripening by regulating chlorophyll degradation and softening in banana. Foods.

[16] Alonso-Salinas R., López-Miranda S., Pérez-López A.J., Acosta-Motos J.R. (2024). Strategies to delay ethylene-mediated ripening in climacteric fruits: Implications for shelf life extension and postharvest quality. Horticulturae.

[17] Soleimani J., Zarrinbal M. (2022). Comparison of the storage effect of straw and some ethylene absorbents in apricot fruit packaging. J. Food Res..

[18] Zhang W., Jiang H., Zhang Y., Cao J., Jiang W. (2021). Synergistic effects of 1-MCP and hot air treatments on delaying softening and promoting anthocyanin biosynthesis in nectarines. Postharvest Biol. Technol..

[19] Zhu Y., Wang K., Wu C., Zhao Y., Yin X., Zhang B., Grierson D., Chen K., Xu C. (2019). Effect of ethylene on cell wall and lipid metabolism during alleviation of postharvest chilling injury in peach. Cells.

[20] Zheng Y., Duan L., Jiang Y., Yang X., Wang H., Li W., Pan N., Wang X., Liang F., Pan Y. (2023). Ozone mitigates the flesh discoloration in response to 1-methylcyclopropene by promoting anthocyanin biosynthesis in postharvest nectarines. Sci. Hortic..

[21] Wei H., Seidi F., Zhang T., Jin Y., Xiao H. (2021). Ethylene scavengers for the preservation of fruits and vegetables: A review. Food Chem..

[22] Tipu M.M.H., Sherif S.M. (2024). Ethylene and its crosstalk with hormonal pathways in fruit ripening: Mechanisms, modulation, and commercial exploitation. Front. Plant Sci..

[23] Lin S., Chen C., Luo H., Xu W., Zhang H., Tian J., Ju R., Wang L. (2019). The combined effect of ozone treatment and polyethylene packaging on postharvest quality and biodiversity of *Toona sinensis* (A.Juss.) M.Roem. Postharvest Biol. Technol..

[24] Goswami M., Mondal K., Prasannavenkadesan V., Bodana V., Katiyar V. (2024). Effect of guar gum-chitosan composites edible coating functionalized with essential oils on the postharvest shelf life of Khasi mandarin at ambient condition. Int. J. Biol. Macromol..

[25] Cui K., Yang L., Shu C., Liu J., Zhu Z., Yang Z., Zhu X., Jiang W. (2021). Near freezing temperature storage alleviates cell wall polysaccharide degradation and softening of apricot (*Prunus armeniaca* L.) fruit after simulated transport vibration. Sci. Hortic..

[26] Wang R., Zhang L., Rahman F.U., Luo J., Liu T., Chen W., Li X., Zhu X. (2024). 1-methylcyclopropene combined with ethylene absorbent delays the ripening of ‘Fenjiao’ banana (Musa ABB Pisang Awak). Sci. Hortic..

[27] Sang X., Yang L., Li D., Xu W., Fu Y., Shi J. (2022). New passive modified atmosphere packaging to extend peaches shelf life at ambient temperature to reduce economic losses. Brit. Food J..

[28] Deng L., Zhou Y., Zeng K. (2015). Pre-harvest spray of oligochitosan induced the resistance of harvested navel oranges to anthracnose during ambient temperature storage. Crop Prot..

[29] Fan X., Jiang W., Gong H., Yang Y., Zhang A., Liu H., Cao J., Guo F., Cui K. (2019). Cell wall polysaccharides degradation and ultrastructure modification of apricot during storage at a near freezing temperature. Food Chem..

[30] Liu X., Xu X., Zhang Y., Xu Y., Chen X., Huang W., Li P. (2025). The MYB transcriptional factor BrMYB108 regulates Auxin-mediated delayed leaf senescence in postharvest Pak Choi. Postharvest Biol. Technol..

[31] Hayat U., Li W., Bie H., Liu S., Guo D., Cao K. (2023). An overview on post-harvest technological advances and ripening techniques for increasing peach fruit quality and shelf life. Horticulturae.

[32] An J., Zhang M., Zhan Z. (2006). Effect of packaging film on the quality of ‘Chaoyang’ honey peach fruit in modified atmosphere packages. Packag. Technol. Sci..

[33] Mahajan B.V.C., Dhillon W.S., Kumar M., Singh B. (2015). Effect of different packaging films on shelf life and quality of peach under super and ordinary market conditions. J. Food Sci. Technol..

[34] Li C., Zhou H., Zhang L., Luo S., Hu H., Li P. (2018). Screening of packaging materials suitable for preservation of peach fruits during shelf-life. Jiangsu Agric. Sci..

[35] Zhang J., Cheng D., Wang B., Khan I., Ni Y. (2017). Ethylene control technologies in extending postharvest ahelf life of climacteric fruit. J. Agric. Food Chem..

[36] Ebrahimi A., Zabihzadeh Khajavi M., Ahmadi S., Mortazavian A.M., Abdolshahi A., Rafiee S., Farhoodi M. (2021). Novel strategies to control ethylene in fruit and vegetables for extending their shelf life: A review. Int. J. Environ. Sci. Technol..

[37] Jacxsens L., Devliegherre F., Van Der Steen C., Siro I., Debevere J. (2003). Application of ethylene adsorbers in combination with high oxygen atmospheres for the storage of strawberries and raspberries. Acta Hortic..

[38] Böhmer-Maas B.W., Fonseca L.M., Otero D.M., da Rosa Zavareze E., Zambiazi R.C. (2020). Photocatalytic zein-TiO_2_ nanofibers as ethylene absorbers for storage of cherry tomatoes. Food Packag. Shelf Life.

[39] Li S., Hu X., Chen S., Wang X., Shang H., Zhou Y., Dai J., Xiao L., Qin W., Liu Y. (2023). Synthesis of γ-cyclodextrin metal-organic framework as ethylene absorber for improving postharvest quality of kiwi fruit. Food Hydrocoll..

[40] Öztürk M., Ayhan Z. (2023). Combined effects of ethylene scavenging-active packaging system and modified atmosphere to reduce postharvest losses of ethylene sensitive produce: Banana and kiwifruit. Packag. Technol. Sci..

[41] Awalgaonkar G., Beaudry R., Almenar E. (2020). Ethylene-removing packaging: Basis for development and latest advances. Compr. Rev. Food Sci. Food Saf..

[42] Wang Y., Lin S., Zhang M., Nie J., Tang A., Sun N., Zeng S., Liu X., Ding Y., Yin X. (2024). Integrated transcriptomic and metabolomic analyses revealed the effect of melatonin on delaying persimmon fruit softening. Postharvest Biol. Technol..

[43] Shi W., Yang H., Jiao J., Wang F., Lu Y., Deng J. (2019). Effects of graft copolymer of chitosan and salicylic acid on reducing rot of postharvest fruit and retarding cell walldegradation in grapefruit during storage. Food Chem..

[44] Wang S., Li L., Zhang Z., Fang Y., Li D., Chen X., Feng S. (2022). Ethylene precisely regulates anthocyanin synthesis in apple via a module comprising MdEIL1, MdMYB1, and MdMYB17. Hortic. Res..

[45] Chen M., Gu H., Wang L., Shao Y., Li R., Li W. (2022). Exogenous ethylene promotes peel color transformation by regulating the degradation of chlorophyll and synthesis of anthocyanin in postharvest mango fruit. Front. Nutr..

[46] Xu Y., Li S.e., Huan C., Jiang T., Zheng X., Brecht J.K. (2020). Effects of 1-methylcyclopropene treatment on quality and anthocyanin biosynthesis in plum (*Prunus salicina* cv. Taoxingli) fruit during storage at a non-chilling temperature. Postharvest Biol. Technol..

[47] Sun H., Hu K., Wei S., Yao G., Zhang H. (2023). Ethylene Response Factors 4.1/4.2 with an EAR motif repress anthocyanin biosynthesis in red-skinned pears. Plant Physiol..

[48] Jayarajan S., Sharma R.R. (2019). Influence of in-package use of ethylene absorbents on shelf life and quality of nectarine during supermarket conditions. Fruits.

[49] Wang Q., Wei Y., Jiang S., Wang X., Xu F., Wang H., Shao X. (2020). Flavor development in peach fruit treated with 1-methylcyclopropene during shelf storage. Food Res. Int..

