# Supplementary material for: Improving the Surface Color and Delaying Softening of Peach by Minimizing the Harmful Effects of Ethylene in the Package
Source: Foods. 2025 Jul 15;14(14):2472. doi: 10.3390/foods14142472 (PMC12295221; doi:10.3390/foods14142472)
Supplement: Supplementary file 1 [file foods-14-02472-s001.zip › foods-3734788-supplementary.pdf]

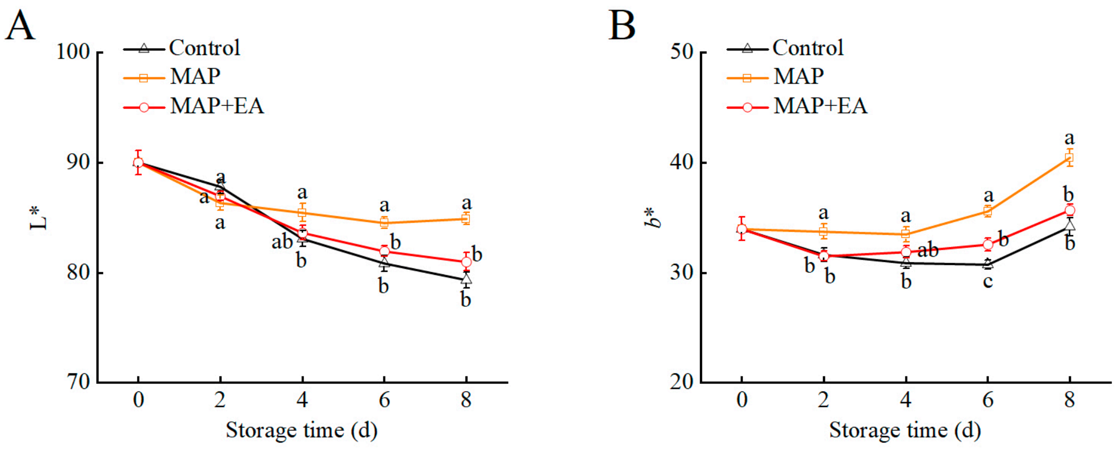

Figure S1: Effect of MAP and MAP+EA treatments on the *L** value (A) and *b** value (B) of peach peel during storage at 25 °C for 8 days.

Table S1 Quantitative Real-time PCR primer sequences for genes associated with anthocyanin metabolism and cell wall degradation

Target gene	Accession number	Forward	Reverse
TEF2	Prupe.4G138700	5'-GGTGTGACGATGAAGAGTGATG-3'	5'-TGAAGGAGAGGGAAGGTGAAAG-3'
PAL	Prupe.6G235400	5'-GTTTGGTGCTACCTCCCACA-3'	5'-AGAGTAGCCCTGGAGGAGTG-3'
CHS	Prupe.1G002900	5'-CAAACCATCCTTCCCGACAG-3'	5'-TTCTCAGGCTTCAGGGCTAAT-3'
CHI	Prupe.2G225200	5'-TGAAGACCTCAAGGAACTTCTCAATGG-3'	5'-ACACAGGTGACAACGATACTGCCACT -3'
F3H	Prupe.7G168300	5'-TCCGAGGGCAGAGCGAAGAAC-3'	5'-TTGTGGAGGCTTGTGAGGATTGG-3'
F3'H	Prupe.5G203600	5'-CCCAACTTGACCTACCTCCA-3'	5'-CTTTGGGATGTGGAAGCTGT-3'
DFR	Prupe.1G376400	5'-GATGCCTGCCGATAGTTCTT-3'	5'-CCCTAACAGTGTAGCCTCTTTC-3'
ANS	Prupe.5G086700	5'-AAGTGGGTCACTGCCAAGTGTGTTCGTC-3'	5'-GTGGCTCACAGAAAACTGCCCAT-3'
UFGT	Prupe.2G324700	5'-GCAAGACTGGTGGAGGACG-3'	5'-GCGAGTAGTTTGACGGTGTTTAT-3'
MYB10.1	Prupe.3G163100	5'-GGATTCTCGCCTGAAAAAGGTG-3'	5'-CGGCGTACTAAAATTCTCGACTG-3'
bHLH3	Prupe.8G242100	5'-TCTTGTTCAGAGTTCCGTTCCT-3'	5'-TTGGCGCTGAGCTCATCTTGTG-3'
WD40	Prupe.2G319500	5'-CCCAGCCTGATACCCCTTTGCT-3'	5'-GTCGGCGAACGGATATCCAAAAT-3'
PG	Prupe.4G261900	5'-TGCAGATGGAACAACTGAGG-3'	5'-GGGCAAACTTTTGGATTTCA-3'
PME	Prupe.7G192800	5'-CATTCCGTCGTGGAACAAGC-3'	5'-CGTAAAAGGTCACGAACGGC-3'
β-Gal	Prupe.4G278500	5'-TAGGACTTTACAAGGGAGA-3'	5'-TTCAAGGTCAATAGAGGG-3'
PLY	Prupe.5G161300	5'-AAAGCCAGGGACTCTACGA-3'	5'-TATGCCCAAGCAACATCA-3'
